# A data driven agent-based model that recommends non-pharmaceutical interventions to suppress Coronavirus disease 2019 resurgence in megacities

**DOI:** 10.1098/rsif.2021.0112

**Published:** 2021-08-25

**Authors:** Ling Yin, Hao Zhang, Yuan Li, Kang Liu, Tianmu Chen, Wei Luo, Shengjie Lai, Ye Li, Xiujuan Tang, Li Ning, Shengzhong Feng, Yanjie Wei, Zhiyuan Zhao, Ying Wen, Liang Mao, Shujiang Mei

**Affiliations:** ^1^ Shenzhen Institute of Advanced Technology, Chinese Academy of Sciences, Shenzhen 518055, Guangdong, People's Republic of China; ^2^ University of Chinese Academy of Sciences, Beijing 100049, People's Republic of China; ^3^ Shenzhen Center for Disease Control and Prevention, Shenzhen 518055, Guangdong, People's Republic of China; ^4^ State Key Laboratory of Molecular Vaccinology and Molecular Diagnostics, School of Public Health, Xiamen University, Xiamen 361102, Fujian, People's Republic of China; ^5^ Geography Department, National University of Singapore, AS2-03-01, 1 Arts Link, Singapore 117570, Republic of Singapore; ^6^ WorldPop, School of Geography and Environmental Science, University of Southampton, Southampton SO17 1BJ, UK; ^7^ National Supercomputing Center in Shenzhen, Shenzhen 518055, Guangdong, People's Republic of China; ^8^ The Academy of Digital China (Fujian), Fuzhou University, Fuzhou 350108, Fujian, People's Republic of China; ^9^ Department of Geography, University of Florida, Gainesville, FL 32611, USA

**Keywords:** COVID-19, agent-based model, contact tracing, facemask, testing, mobile phone data

## Abstract

Before herd immunity against Coronavirus disease 2019 (COVID-19) is achieved by mass vaccination, science-based guidelines for non-pharmaceutical interventions are urgently needed to reopen megacities. This study integrated massive mobile phone tracking records, census data and building characteristics into a spatially explicit agent-based model to simulate COVID-19 spread among 11.2 million individuals living in Shenzhen City, China. After validation by local epidemiological observations, the model was used to assess the probability of COVID-19 resurgence if sporadic cases occurred in a fully reopened city. Combined scenarios of three critical non-pharmaceutical interventions (contact tracing, mask wearing and prompt testing) were assessed at various levels of public compliance. Our results show a greater than 50% chance of disease resurgence if the city reopened without contact tracing. However, tracing household contacts, in combination with mandatory mask use and prompt testing, could suppress the probability of resurgence under 5% within four weeks. If household contact tracing could be expanded to work/class group members, the COVID resurgence could be avoided if 80% of the population wear facemasks and 40% comply with prompt testing. Our assessment, including modelling for different scenarios, helps public health practitioners tailor interventions within Shenzhen City and other world megacities under a variety of suppression timelines, risk tolerance, healthcare capacity and public compliance.

## 1. Introduction

Coronavirus disease 2019 (COVID-19) outbreaks have increased in several countries after initial strict restrictions on businesses, schools and public life were lifted [1–4]. Many governments are now faced with a dilemma between socio-economic recovery and disease resurgence [5,6]. Before herd immunity is achieved by long-term mass vaccination, evidence-based guidelines to minimize the risk of resurgence are urgently needed for post-epidemic management [7–11].

COVID-19 incidence in countries with high mask usage and effective contact tracing (e.g. China and Singapore) is much lower than that in countries with insufficient compliance with regard to facemasks and/or with insufficiently comprehensive contact tracing (e.g. the United States and Spain) [12,13]. Prior experience as well as empirical studies have demonstrated the importance of contact tracing and facemask wearing in reducing the transmission of COVID-19 [1,14–18]. Moreover, prompt testing after symptom onset can not only shorten transmission periods but also improve the effectiveness of contact tracing [19]. However, few studies have examined the likelihood of resurgence under various combined interventions of contact tracing, facemask wearing and prompt testing interventions.

Megacities with high concentrations of people (more than ten million), businesses and schools are most affected by COVID-19 in terms of infection rate, mortality rate and economic loss [20]. The transmission of COVID-19 in a megacity is highly complex and nonlinear due to daily movements of millions of individuals with diverse demographics, various types of their activities (e.g. at home, work, study and leisure) within a heterogeneous environment, and dynamic contacts formed during these activities [21,22]. Modelling such realism requires various datasets at fine spatio-temporal resolutions, such as mobile phone tracking records, census block data and building locations [23–25]. To the best of our knowledge, most COVID-19 simulation models built for megacities have not yet achieved this level of realism [1,26], and few models have been validated based on local observations of COVID-19 evolution [27]. This lack of validation may reduce the transferability of suggested interventions to operational guidelines within public health practice.

Furthermore, most COVID-19 models are limited to comparing relative differences between various interventions [1,28–31], and little attention has been paid to assessing the probability of COVID-19 resurgence in complex urban systems [27]. Small probability events, such as super spreading, could occur in these complex transmission systems because of nonlinear effects. To manage the risk of disease resurgence, outbreak probabilities under different conditions are as important as the size of the epidemic [18,28,32,33].

This study developed a data-driven, individual-activity-based model to simulate COVID-19 transmission for the entire population of Shenzhen City, China. This spatially explicit agent-based model integrated multiple real datasets, including the mobile phone trajectory records of 5.8 million users, inter-city flows from Baidu migration data, a travel survey of approximately 98 000 households, a database of 0.6 million buildings, social contact surveys and the census data. The model simulated the spatial spread of COVID-19 among approximately 11 million individuals moving between nearly five million activity places (home, workplace, school and public places). After calibration and validation by the first wave epidemic data, the model was used to assess the probability of disease resurgence during the post-epidemic period if sporadic cases occurred in a fully reopened city under various combined interventions of contact tracing (household, workplace, school or public), mask wearing and prompt testing. The findings from our study could help public health practitioners tailor practical guidelines for megacities, considering different suppression timelines, minimum and maximum levels of risk allowed, healthcare capacity, general infrastructure and public compliance with control measures.

## 2. Methods

### 2.1. A spatially explicit agent-based system

**Figure 1.**
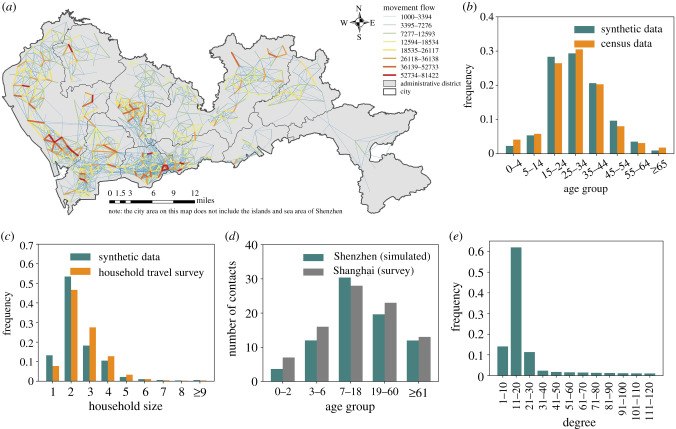
Demographic characteristics, movement flows and contact networks in the spatially explicit agent-based model. (*a*) Intra-urban movement flows of mobile phone users between cell towers, derived from mobile phone trajectory records. (*b*) Age composition and (*c*) household size of the synthetic population as compared to actual census data and household travel survey. (*d*) The simulated average daily contacts per person by age group, compared to that observed in Shanghai. (*e*) Degree distribution of the simulated daily contact network.

We first used a data-fusion algorithm to synthesize 11.2 million residents (i.e. agents) with demographic characteristics. This was accomplished by cross-referencing census data and household travel survey [24]. We then assigned hourly movements of these residents among 0.6 million locations (e.g. residential buildings, schools, offices and restaurants) (electronic supplementary material, figure S1): for mobile phone users, we used mobile phone trajectory records (figure 1*a*); for non-mobile phone users, we based their movements on the household travel survey. As a result, each individual was anchored onto different types of buildings as places for living, working, studying and performing other activities, forming a daily travel trajectory. Individuals allocated to the same buildings were grouped into 4.5 million households (figure 1*b*,*c*) and 230 000 workplaces (including schools). The model allowed the synthesized individuals to have contact with one another when staying at the same location within one hour, resulting in a spatially explicit and temporally dynamic network of contacts that could spread COVID-19. To further model the populations travelling into and out of the city [34], we used Baidu migration data as a reference (electronic supplementary material, figure S2) and randomly selected a number of individuals to leave the system at one day and then return back at another day [35]. More details of population synthesis and network construction can be found in the electronic supplementary material.

We simulated *close contacts* as well as *casual contacts* to represent diverse human contact patterns. The close contacts represented regular encounters with a small group of acquaintances in households, workplaces, and schools and daycares, while the casual contacts represented random encounters with strangers at various public places, such as shops and restaurants [36,37]. Between these two contact patterns, individuals' mixing patterns and contact intensities were varied (see the electronic supplementary material for more details). The daily contact network was calibrated to follow the degree distribution observed in Shanghai (figure 1*d,e*), a Chinese megacity of equivalent scale [38].

#### 2.1.1. Epidemic simulation

**Figure 2.**
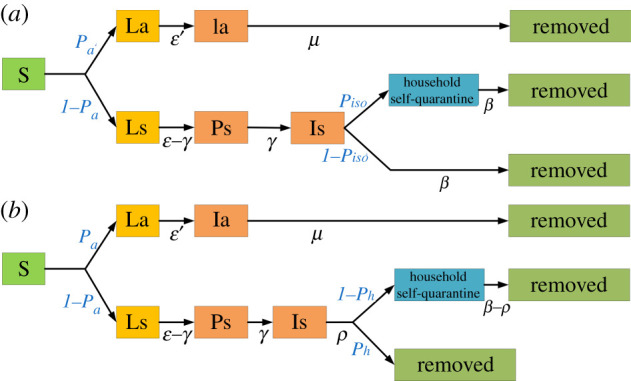
A compartmental model of COVID-19 for (*a*) the baseline scenario and (*b*) the post-epidemic period.

We implemented a stochastic, discrete-time susceptible–latent–infectious–removed (SLIR) model, in which the transmission of COVID-19 was triggered by contacts between individuals in households, workplaces, schools and other buildings. Once a susceptible individual had a contact with an infectious individual, the probability of infection *p* via this contact was calculated as follows:

2.1 $$p=\mathrm{pTrans}\times I_{c}\times r,$$

where pTrans denotes the transmission probability per contact and was estimated as 0.165 by calibrating the modelled basic reproductive number *R*_0_ to the observed value of 2.4 [1,32,39–41]. *I_c_* is the intensity of daily contact at different contact settings derived from a contact survey of Shanghai (electronic supplementary material, table S1) [38]; *r* differentiates the infectivity of infectious individuals with and without symptoms, i.e. the infectivity of asymptomatic individuals was set as 0.12 of their counterparts [42]. Our simulations assumed that all infected individuals would not be re-infected. As shown in figure 2 and electronic supplementary material, table S2, once a susceptible individual (S) was infected, we assumed a 25% probability (*P_a_*) of turning into latent status [33,43,44]. The latent period (La) was set to 4.6 days (*ɛ’*) for asymptomatic individuals to become infectious (Ia) [45–47]. These individuals remained infectious for 9.5 days (*μ*) until being removed from the model after recovery or being centralized quarantined [48]. Symptomatic individuals were assigned an incubation period (*ɛ*) with a mean of 5.2 days to manifest symptoms (Is), including latent status (Ls) [47]. Their infectivity started from 2 days (*γ*) before symptom onset (Ps) [46]. After the onset of symptoms, individuals remained infective until they were removed by interventions such as hospitalization or centralized quarantine.

Note that some of the SLIR model parameters were fixed values while some others were derived from probability distributions. In addition to the parameters reported in the existing studies, the major rationale of our choice between fixed values and distributions was based on the assessment of our local Center for Disease Control (CDC) collaborators, who had first-hand COVID-19 clinical data. If they were confident on the parameter estimates, we used fixed values; otherwise, we used distributions.

### 2.2. Simulation of interventions

During the first wave of COVID-19 in Shenzhen (from 1 January to 1 March 2020), five types of intervention strategies were applied by the city government, namely comprehensive isolation and quarantine measures, prompt testing, mask wearing, social distancing and city lockdown. A detailed description of how these five intervention strategies were incorporated into our simulation model can be found in the electronic supplementary material, table S3. After the first wave, four of them remained effective to prevent a resurgence and are briefly described below since they are the focus of our study.

#### 2.2.1. Prompt testing

People with COVID-19-like symptoms were urged to see doctors at the earliest possible time, where they received nucleic acid tests for COVID-19 diagnosis. Once a positive test result was confirmed, the infected individual was immediately isolated for medical treatment and removed from the model.

#### 2.2.2. Contact tracing

Close contacts of a confirmed case were quarantined at specific hotels for 14 days, and they were not allowed any in-person contact during the quarantine. After 14 days, those uninfected individuals were released, while those who were newly diagnosed initiated another round of contact tracing. In our model, the quarantine was implemented immediately after contact tracing. We did not consider the time delay in contact tracing due to the local situation in Shenzhen [15,49]. Once the infected individuals were diagnosed, the local government immediately traced their close contacts in households and workplaces, and in most cases, this procedure would be completed within 24 h.

#### 2.2.3. Mask wearing

Wearing masks for outdoor activities was strongly recommended, but not mandated, by the city government. For the use of facemasks, the probability of infection per contact was adjusted using the following equation:

2.2 $$p=\mathrm{pTrans}\times I_{c}\times r\times (1-\theta ),$$

where *θ* is the effectiveness of wearing masks.

#### 2.2.4. Self-quarantine at home

Individuals with COVID-19-like symptoms voluntarily quarantined at home and no longer engaged in outdoor activities, even before their infection was confirmed. During a quarantine period, the self-quarantined person only had contact with household members, and the transmission to other household members was reduced by *δ*, referred to as the effectiveness of household self-quarantine. The probability of transmission per contact was adjusted as follows:

2.3 $$p=\mathrm{pTrans}\times I_{c}\times r\times (1-\delta ).$$

### 2.3. Baseline scenario

The baseline scenario represents the actual course of the first epidemic in Shenzhen City and provides a basis for our post-epidemic simulation. We introduced a few imported cases on the date of their arrival in Shenzhen, according to data from the local CDC and Prevention report. For each imported case, we synthesized a new household based on reported age, gender, household structure and residence. We then randomly selected an existing synthesized household with the same characteristics and assigned all travel trajectories from this household to the imported case as well as to the other household members. In addition, we simulated the five interventions mentioned above to mimic the actual control efforts of the local government during this time period, as detailed in the electronic supplementary material, table S3, and elaborated in the electronic supplementary material.

We simulated the baseline scenario for 1000 realizations. A video of one model realization can be found in the electronic supplementary material. We generated the averaged epidemic curve and calibrated model parameters in the electronic supplementary material, table S3, by fitting the resulting curve to actual cumulative symptomatic cases reported by the local health agency. The effectiveness of the household self-quarantine variable *δ* was fitted to a value of 0.7, while the effectiveness of mask wearing *θ* ranged from 0.5 to 0.9 (see more details in the electronic supplementary material). To validate the model outcomes, we examined the spatial and age distributions of symptomatic cases as well as the household secondary attack rate.

### 2.4. Intervention scenarios for the post-epidemic period

To evaluate the risk of future outbreaks, we extended the baseline model to simulate a post-epidemic period after 18 May 2020, when people's fear of the disease gradually faded and schools started to reopen. To initiate the next outbreak from sporadic cases, we randomly selected 30 individuals as infective seeds. These individuals worked at five different buildings in crowded areas and had an above-average daily contact number. We randomly set 25% of the seeds to be asymptomatic.

We focused on three types of interventions that are most concerning to local policymakers: contact tracing, mask wearing and prompt testing after symptom onset. We considered five levels of contact tracing: Level 0 as no tracing; Level I only traced household members; Level II traced all close contacts in the same household and workgroup/class; Level III traced all close contacts and 50% casual contacts in public places; lastly, Level IV as 100% contact tracing, including all close contacts and casual contacts. For the use of masks and prompt testing, we varied compliance levels from 0 to 100% with a 20% increment. Within figure 2*b* and electronic supplementary material, table S2, we assumed that symptomatic cases (if compliant with the government policy of prompt testing) took a nucleic acid test within 2 days after symptom onset [1,50]; otherwise, they would quarantine themselves at home (due to the prevalent symptoms of fever) until testing [15,16]. We assumed that symptomatic cases would eventually be tested due to the increasing convenience of testing and that there was no delay between testing and disease confirmation. To account for different types of facemasks, we considered low (50%) and high (70%) protective effectiveness [14,17]. In total, we evaluated 360 (=5 × 6 × 6 × 2) combinations of interventions. We simulated each combination for 100 days for 1000 realizations and estimated the effective reproductive number *R_t_* for each realization. We defined disease resurgence as *R_t_* > 1 at the end of *t* weeks since the first symptomatic case occurred. The probability of disease resurgence was calculated from 1000 realizations. We set 5% as a socially acceptable threshold for successful suppression. For each combination above, if the resurgence probability could be reduced by less than 5% within four weeks (*t* = 4), we considered this to be a fast suppression. If eight weeks (*t* = 8) were needed, we termed this a slow suppression.

## 3. Results

This model was programmed in Python 3.6.4 and implemented in a high-performance computing cluster environment. The high-performance computing cluster we used had 836 CPU cores and a single node with 192G of memory.

### 3.1. Validation of the baseline model

**Figure 3.**
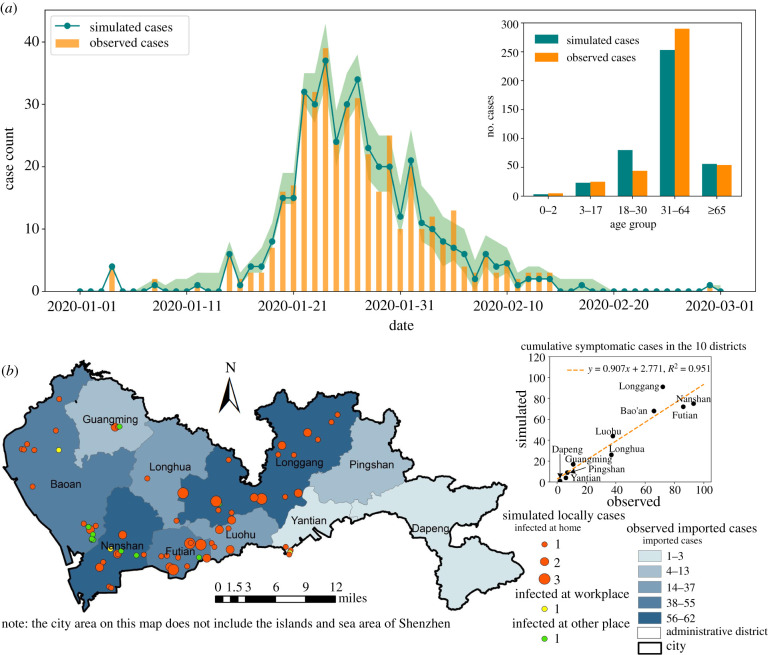
The simulated results for the first wave of COVID-19 in Shenzhen as the baseline scenario. (*a*) The simulated daily new symptomatic cases as compared to local CDC reported cases. The green shaded area indicates the 95% confidence interval. The inset compares the age distribution of simulated cases to the observed data. (*b*) Spatial distribution of observed imported cases and simulated local infections. The scatterplot compares the simulated and observed symptomatic cases by district in one model realization.

The baseline scenario model yielded an average of 416 symptomatic cases in the epidemic, which was close to the 418 cases confirmed by the local CDC. We estimated a low root mean squared error (RMSE = 1.35) between the simulated and observed daily cases, indicating that the simulated epidemic curve matched well with the observed data in terms of both magnitude and timeline (figure 3*a*). Over each age group, the predicted symptomatic cases showed a high degree of agreement with the confirmed cases. Most locally acquired infections occurred within households in our simulation, which was similar to data within actual observations [15]. On average, the simulated household secondary attack rate was 11.02%, which is in line with other observational studies [15,16]. Our model also achieved a high spatial accuracy at the administrative district level (figure 3*b*), as the predicted symptomatic case numbers were highly consistent with reported case numbers (*R*^2^ = 0.95).

### 3.2. Effectiveness of interventions against disease resurgence

**Figure 4.**
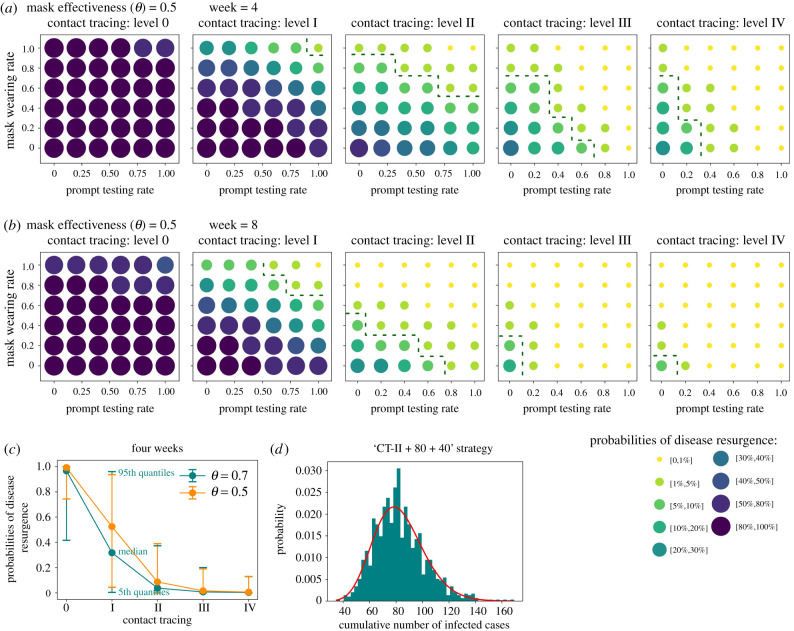
(*a*,*b*) Probabilities of disease resurgence under various combinations of three interventions. The green dashed curve is a contour line of 5% threshold, below which the disease resurgence is considered suppressed. Results are shown with a suppression period of (*a*) four weeks and (*b*) eight weeks. (*c*) Comparison of the effect of different levels of contact tracing. (*d*) Distribution of cumulative symptomatic cases within the first 100 days averaged from 1000 realizations of the ‘CT-II + 80 + 40’ strategy recommended for Shenzhen. It roughly follows a gamma distribution (red solid line) with shape = 19.23 and scale = 4.297, implying the existence of super-spreading events.

The baseline model was extended to estimate the probability of COVID resurgence occurring when compliance to control interventions gradually recedes. Figure 4 and electronic supplementary material, figure S8, show how combinations of interventions suppress disease resurgence given low and high mask effectiveness. Both scenarios suggest that the risk of disease resurgence is most sensitive to levels of contact tracing, followed by individuals' compliance with mask wearing, and is least sensitive to compliance with prompt testing. None of these interventions can suppress outbreak resurgence on their own within four weeks, and thus a combined manner is needed.

In a scenario when a low-effectiveness mask was used by the public (figure 4*a*), we found that Level I contact tracing (i.e. contact tracing of household members) must be applied with mandatory (100%) mask use and prompt testing to achieve fast suppression (i.e. a resurgence probability of under 5% within four weeks). If contact tracing can be expanded to work/class group members (Level II contact tracing), public compliance with mask use and prompt testing can be relaxed to 80% and 40%, respectively. More comprehensive contact tracing that includes casual contacts (Level III and IV contact tracing) could lower the public compliance level for the other two interventions to zero. However, figure 4*c* indicates that Level II contact tracing appears to be a threshold for disease control, in that more labour-intensive contact tracing efforts (such as Level III and IV) do not significantly change the probability of disease resurgence.

An improvement in mask effectiveness to 0.7 and the extension of the suppression period to eight weeks (electronic supplementary material, figure S8b) further relaxed the requirements for public compliance with mask use and prompt testing, offering more possible combinations of the three interventions to prevent disease resurgence. However, compliance with mask wearing cannot be guaranteed and the effectiveness of mask wearing varies with mask type and contact setting [14,17]. Therefore, this study will focus on results for lower mask effectiveness (0.5) in the discussion, thus presenting modelling considerations given a minimum level of control effort.

## 4. Discussion

Our validated model provides a reliable virtual platform for predicting the odds of COVID-19 resurgence after megacity reopening as well as tuning non-pharmaceutical interventions to minimize the risk of disease resurgence. Here, we demonstrated how prompt testing, contact tracing strategies at scale and compliance with facemask use have the potential to provide a viable course of action to mitigate the epidemic after efforts of non-pharmaceutical interventions gradually diminish. Our simulations suggest a ‘CT-II + 80 + 40’ strategy (i.e. contact tracing Level II + 80% compliance with mask use + 40% prompt testing) as the minimum level of control effort for Shenzhen and possibly other megacities in China that require rapid suppression of sporadic COVID-19 outbreaks (figure 4*a*). Due to the high percentage of mild symptomatic COVID-19 cases, particularly in the working population, it is challenging to maintain high-level population compliance to prompt testing after symptom onset, as the fear of COVID-19 has simultaneously been receding in China [15,51,52]. In practice, the local health agency is expected to spare no effort to trace close contacts within households, workplaces and schools for any confirmed case, and to ensure their quarantine for a designated time period. The government should make efforts to normalize mask use in public life and mandate face covering in enclosed spaces, such as public transit, workplaces and restaurants, until vaccines are widely manufactured and distributed. Meanwhile, to ensure a minimum compliance rate of 40% to prompt testing after onset, extensive temperature checks should be implemented in public places to identify people with fever and limit their out-of-home activities. Paid sick leave can be granted to sick people who are willing to take a nucleic acid test as compensation for their loss of productivity [52].

Our model also sheds light on combating COVID-19 in other world megacities. Different cities can customize appropriate levels of contact tracing and compliance rates when assessing their own healthcare capacity. For large cities in European and Asian countries, stringent contact tracing (e.g. Level II and above, with the aid of mobile apps) and high rates of mask use (e.g. 80%) are likely to be maintained [37]. The ‘CT-II + 80 + 40’ strategy could be a feasible option to suppress disease resurgence. For many cities in the United States, where the use of masks remains controversial and public compliance is relatively low, the ‘CT-II + 60 + 0’ or ‘CT-II + 40 + 20’ strategy could be a practical solution to minimize the risk of resurgence within eight weeks (figure 4*b*) [53]. Some large cities in developing countries may have limited manpower and health resources to maintain a high level of contact tracing and a high compliance rate of prompt testing, whereas a mandatory order of mask use and household self-quarantine is more feasible. These cities could set up control strategies as a combination of ‘CT-I + 100 + 0’ or ‘CT-I + 80 + 20’ (figure 4*b*), which can confine the chance of disease resurgence between 5% and 20% within eight weeks.

The three single interventions we simulated for post-epidemic management have been investigated separately by existing studies. First, contact tracing has been widely recommended by many studies [18,19,37]. Particularly, the study in the UK showed that contact tracing of acquaintances alone could have an effect on transmission reduction similar to that of detailed contact tracing [18], which is consistent with our finding that labour-intensive contact tracing efforts for casual contacts do not significantly change the probability of disease resurgence. Second, we recommend 80% of the population to wear masks, which is consistent with the study in New York that also prompted a near universal adoption (80%) of moderately effective (*θ* = 50%) masks [54]. Third, in terms of prompt testing, a recent study has demonstrated that minimizing testing delay had a significant impact on reducing onward transmissions [37]. Compared with the above research, this study explored optimal combinations of the three single interventions to suppress sporadic cases in a fully reopened megacity.

This study has several limitations. First, our mobile phone trajectory records cover only one typical weekday. We did not differentiate between weekday and weekend activities in the model simulation, particularly after reopening. Since people tend to have closer contact on weekdays than on weekends, we believe our model simulated a worse scenario of reopening than the reality and thus the suggested policies should remain reasonable and effective. Second, our understanding of COVID-19 is still improving, and many disease parameters in this model are still inconclusive, such as virus infectivity among various age groups, the proportion of asymptomatic cases and their infectivity as compared to symptomatic cases. Our model parameterization was mainly based on local and national CDC reports. Therefore, the adoption of these disease parameters should be at the discretion of other researchers.

## 5. Conclusion

The contribution of this study to the literature is twofold. First, our model offers a data-driven, fine-grained, agent-based and spatio-temporally resolved presentation of COVID-19 spread in a Chinese megacity. This model accounts for spatio-temporal heterogeneity and the uncertainty of COVID-19 transmission in a complex urban environment, including possible super-spreader transmissions (figure 4*d*). The model was further validated by the intra-urban spatial distribution of cases, the age distribution of cases and household secondary attack rate. We are not aware of any previous megacity simulation that achieved a similar level of realism and reliability. Second, we not only estimated the scale of the COVID-19 outbreak, but also focused on assessing the probability of future outbreaks in Shenzhen City. We explored the complex associations between the probability of disease resurgence and the combination of different contact tracing levels (household, workplace, school and public places), mask wearing rates and prompt testing rates, thus offering policy options for post-epidemic management for megacities of different countries.

As COVID-19 resurges in many countries, megacities face the same issue as Shenzhen in balancing disease risk and economic reopening. For long-term disease control and prevention, intense forms of travel restriction (such as city lockdowns and stay-at-home orders) are not feasible strategies to fight against COVID-19 due to their associated socio-economic disruption. Our model advocates contact tracing of close contacts in household, workplace and school settings, along with high compliance with mask wearing as a priority for the city's post-epidemic management. These results offer public health practitioners within megacities worldwide valuable insights for preventing disease resurgence before herd immunity is achieved by mass vaccination.

## Data accessibility

Mobile phone data were provided by the Shenzhen Transportation Operation Command Center (Contact: Binliang Li, 240854198@qq.com). Travel survey data, building survey data and census data were offered by the Planning and Natural Resources Bureau of Shenzhen Municipality (Contact: Renrong Jiang, jiangrenrong@126.com). The epidemic surveillance data were provided by the Shenzhen Center for Disease Control and Prevention (Contact: Shujiang Mei, sjmei66@163.com). Researchers who meet the criteria for access to confidential data can send requests to the above local government departments. The daily confirmed cases of COVID-19 are publicly accessible from the Shenzhen Municipal Health Commission (http://wjw.sz.gov.cn/yqxx/). Baidu migration data can be openly obtained from http://qianxi.baidu.com/.
